# Helical antimicrobial peptides assemble into protofibril scaffolds that present ordered dsDNA to TLR9

**DOI:** 10.1038/s41467-019-08868-w

**Published:** 2019-03-04

**Authors:** Ernest Y. Lee, Changsheng Zhang, Jeremy Di Domizio, Fan Jin, Will Connell, Mandy Hung, Nicolas Malkoff, Veronica Veksler, Michel Gilliet, Pengyu Ren, Gerard C. L. Wong

**Affiliations:** 1 Department of Bioengineering, University of California, Los Angeles, Los Angeles, CA 90095 USA; 2 Department of Biomedical Engineering, The University of Texas at Austin, Austin, TX 78712 USA; 3 Department of Dermatology, Lausanne University Hospital CHUV, 1011 Lausanne, Switzerland; 4 Hefei National Laboratory for Physical Sciences at the Microscale, Department of Polymer Science and Engineering, CAS Key Laboratory of Soft Matter Chemistry, University of Science and Technology of China, Hefei, 230026 PR China; 5 College of Chemistry and Molecular Engineering, Peking University, Beijing, 100871 PR China

## Abstract

Amphiphilicity in ɑ-helical antimicrobial peptides (AMPs) is recognized as a signature of potential membrane activity. Some AMPs are also strongly immunomodulatory: LL37-DNA complexes potently amplify Toll-like receptor 9 (TLR9) activation in immune cells and exacerbate autoimmune diseases. The rules governing this proinflammatory activity of AMPs are unknown. Here we examine the supramolecular structures formed between DNA and three prototypical AMPs using small angle X-ray scattering and molecular modeling. We correlate these structures to their ability to activate TLR9 and show that a key criterion is the AMP’s ability to assemble into superhelical protofibril scaffolds. These structures enforce spatially-periodic DNA organization in nanocrystalline immunocomplexes that trigger strong recognition by TLR9, which is conventionally known to bind single DNA ligands. We demonstrate that we can “knock in” this ability for TLR9 amplification in membrane-active AMP mutants, which suggests the existence of tradeoffs between membrane permeating activity and immunomodulatory activity in AMP sequences.

## Abstract (web summary)

Amphihelical antimicrobial peptides (AMPs) are bactericidal host defense factors, but their function as immunomodulators is emerging. Here the authors show that several AMPs organize DNA into periodic nanocrystals by self-assembling into superhelical protofibril scaffolds, which potentiates DNA sensing by TLR9.

## Introduction

Antimicrobial peptides (AMPs) are multifunctional molecules important for host defense^1^. Many AMPs are amphiphilic ɑ-helices (AaHs), and can kill pathogens by permeating membranes and inhibiting intracellular processes^1–3^. However, evidence indicates that AMPs can also be immunomodulatory, inducing cytokine production, chemoattraction, and immune cell differentiation^4–7^. Recently, it has been shown that AMPs can modulate Toll-like receptor (TLR) activation by endogenous and microbial immune ligands such as lipopolysaccharide^8^ and nucleic acids like DNA and RNA^9^, and that complex formation between AMPs and immune ligands may be important. Normal expression of AMPs results in immunostimulation that potentially synergizes with direct antimicrobial activity in immune responses to microbial invasion, but aberrant expression of AMPs can lead to uncontrolled inflammation in autoimmune diseases like systemic lupus erythematosus and psoriasis^10–15^. Specifically, modulation of TLR9 signaling in plasmacytoid dendritic cells (pDCs) leads to amplification of type I interferon (IFN) production, which drives disease progression^4^. In a more general compass, although ɑ-helical motifs are common, only a small subset exhibits this proinflammatory activity. At present, we do not know the fundamental rules governing how ɑ-helical motifs assemble with immune ligands like DNA into structural states that enable TLR9 modulation.

In a parallel development, there has been significant interest in understanding the programmed assembly of chemically patchy, anisotropic objects into structures beyond the well-known amphiphilic assembly of surfactants into micelles^16^. Janus particles are nanoparticles with surfaces that have two or more distinct properties that can be changed by varying the material or surface chemistry, which modulate the particle’s local charge, hydrophobicity, dipole moment, and polarizability. The inherent asymmetry in the surface properties of these particles leads to complex effects such as the self-assembly of colloids with directional bonding^17^ and programmed assembly of Janus particles into a Kagome lattice^18^. Machine learning on particle tracking data of Janus particles reveals unique self-assembly pathways involving formation of clusters, chains, and pinwheels^19^. Assembly of peptides into fibrils has long been a hallmark of amyloid diseases^20^, and recently self-assembly of peptide amphiphiles into ordered structures has been shown to be important for regenerative medicine^21,22^. For assembling units with more complex geometry than surfactants, shape and chemical heterogeneity can exact competing demands on the assembly process and lead to counterintuitive results^23^. Even without the complications from amphiphilic self-assembly, cationic globular molecules and anionic biological polyelectrolytes are known to order into columnar electrostatic complexes with structural polymorphism^24–26^. Although AMPs are known to aggregate under certain conditions^27^, it is not clear from the perspective of self-assembly of anisotropic Janus objects how curved, cationic AaHs will electrostatically assemble with anionic DNA, much less how the geometry of the resultant self-assembled AaH-DNA structures connect to their immunomodulatory behavior. Elucidation of these structures is complicated by the fact that immunologically relevant complexes tend to be small enough for endocytosis (<~200 nm as measured by dynamic light scattering) and weakly ordered due to their macromolecular nature, hence not optimal for crystallography.

Here we examine the structures of immunologically active complexes formed between DNA and three prototypical ɑ-helical AMPs (melittin, LL37, and buforin) and related mutants from the perspective of Janus self-assembly using a combination of synchrotron small-angle X-ray scattering (SAXS) and molecular modeling. By correlating these structures to IFN production by human pDCs and by mouse macrophages, we find that a key criterion is the Janus peptide’s ability to function as subunits that assemble into superhelical protofibrils with hydrophobic cores in the presence of DNA, which is possible with curved but not straight helices. In order to maximize the entropy gain of counterion release, these cationic coiled-coil protofibrils interact with anionic DNA to form columnar protofibril-DNA nanocrystals. These AMP-DNA complexes are then internalized into the endosomes of immune cells like macrophages and dendritic cells and are strongly recognized by TLR9^28^. TLR9 is conventionally thought to bind single naked double-stranded DNA (dsDNA) ligands^29^. However, we find that periodic aggregates of nanocrystalline DNA ligands scaffolded by AMPs amplify TLR9-mediated inflammation by enhancing TLR9 recruitment and binding through multivalent effects. The formation of such AaH-DNA nanocrystals explains how AMPs can organize DNA into spatial periodicities that match the steric size of TLR9^28,30^, which is not possible without higher-order protofibril self-assembly. Interestingly, the AaH protofibrils can adapt their helical pitch to optimize binding: protofibrils of melittin and those of LL37 have pitches that differ by a factor of ~2, in order to preserve the same density of positive charges for optimal DNA charge compensation. Using AMPs melittin, LL37, and buforin, we show how AaH hydrophobicity impacts protofibril self-assembly, which track with the level of TLR9 activation. Interestingly, melittin-related peptides with systematic reduction of membrane remodeling activity measured with SAXS led to a progressive increase of TLR9-activating ability. These observations suggest that the requirements for membrane activity and immunomodulation via TLR activation, two canonical functions of AMPs which both require a facially amphiphilic helix architecture, are overlapping but clearly distinct. Taken together, these results provide insight into trade-offs between membrane activity and immunomodulatory activity in AMPs.

## Results

### Selection of ɑ-helical AMPs

To understand dsDNA condensation and immune stimulation by AaH, we selected three prototypical AMPs. LL37 is an ɑ-helical AMP and has been previously shown to enable immune activation in lupus and psoriasis by forming complexes with dsDNA^10,11,13^. LL37 (+6 charge at pH 7.4) is a low-symmetry object; it has a curved amphipathic helix-bend-helix motif with a glycine at position 14. We hypothesized curved ɑ-helices with compensating cationic charge density and amphipathicity may assemble into a macromolecular scaffold to organize dsDNA. We chose melittin and buforin as direct comparisons to LL37 since they are amphiphilic but have different charge densities and hydrophobicities, with little sequence similarity. Full details of peptide selection and sequence analysis are found in the Supplementary Discussion. Using these AMPs, we correlate self-assembled AMP-dsDNA structures with their ability to activate TLR9.

### Melittin organizes dsDNA into a tetragonal lattice

**Fig. 1:**
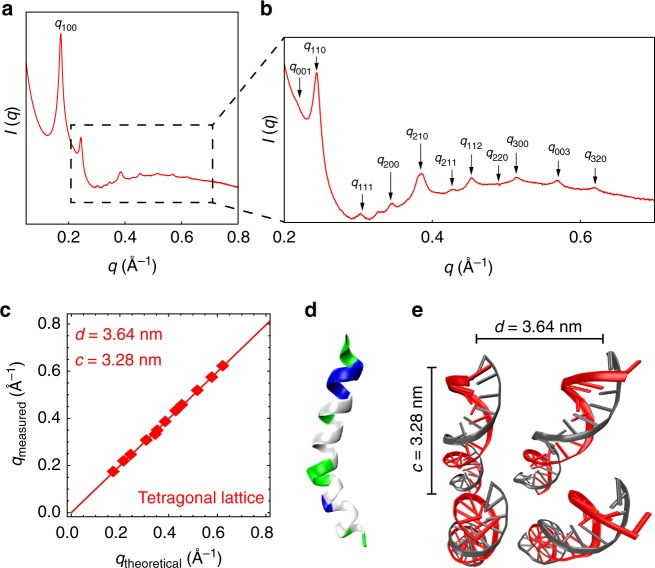
Small-angle X-ray scattering (SAXS) structure of melittin-double-stranded DNA (dsDNA) complexes. **a** Representative SAXS data of an isoelectric melittin-dsDNA complex with the first Bragg peak *q*_100_ indicated. **b** The magnified data region shows details of diffraction features *q*_*hkl*_, which index well to a three-dimensional tetragonal lattice. **c** To obtain the lattice parameters, we fit the measured Bragg peak positions to predicted reflections using multiple linear regression. The lattice parameters of the melittin-dsDNA complex are *d* = 3.64 nm and *c* = 3.28 nm (*R*^2^ = 0.999). **d** Structure of the melittin monomer (PDB: 2MLT_A^57^). Cationic residues are colored blue, hydrophobic residues are colored white, and polar uncharged residues are colored green. **e** Schematic of parallel dsDNA rods within the melittin-dsDNA tetragonal lattice labeled with lattice parameters obtained from SAXS. The average domain size of the melittin-dsDNA lattice is *L* ~ 62.4 nm

**Fig. 2:**
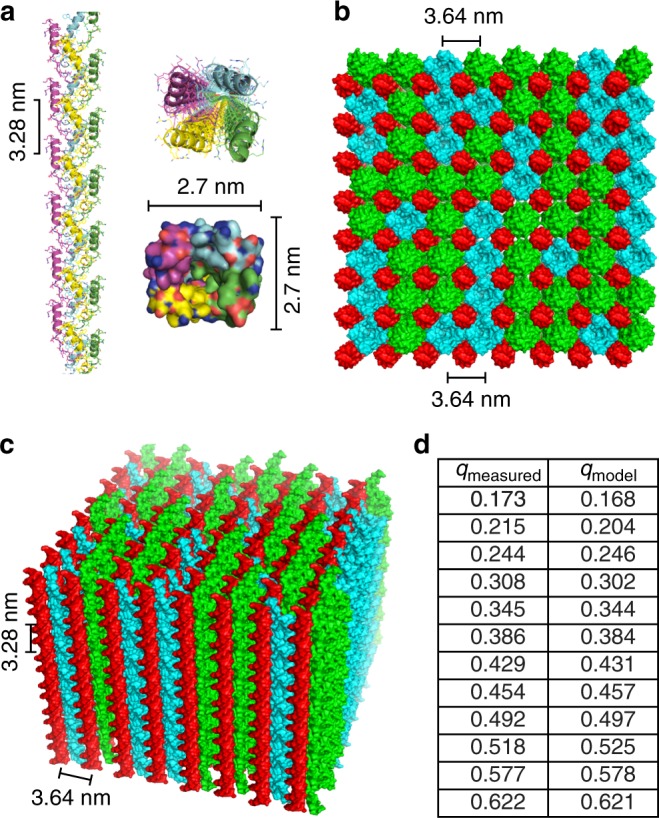
Structure of melittin-double-stranded DNA (dsDNA) complexes from molecular simulations. **a** Side and top views of the melittin protofibril self-assembled in the presence of dsDNA. The cross-sectional dimensions of the protofibril is 2.7 nm × 2.7 nm. The helical pitch of the protofibril is 3.28 nm per turn. Top (**b**) and side (**c**) views of an 8 × 8 melittin-dsDNA square lattice from molecular dynamics simulations. Columnar dsDNA is colored red, and the melittin protofibril is colored green (N to C terminus polarity) and teal (C to N terminus polarity). Random polarity of the melittin protofibril is assumed. The inter-dsDNA spacing from the molecular model obtained from simulation is *d* = 3.64 nm, which agrees well with the predicted inter-dsDNA spacing from small-angle X-ray scattering (SAXS). The out-of-plane lattice parameter of *c* = 3.28 nm also agrees with the simulated pitch of the melittin protofibril. **d** There is good agreement between the observed structure factor peaks from SAXS with peaks recovered from theoretical X-ray scattering of the lattice model in **b** and **c** from CRYSOL^58^

We investigate the structure of melittin-dsDNA complexes using a combination of SAXS and molecular modeling. From SAXS, we find that solubilized DNA alone does not form ordered structures (Supplementary Figure 1). In contrast, melittin-DNA forms a three-dimensional (3D) tetragonal lattice with Bragg reflections at *q*_100_ = 0.173, *q*_001_ = 0.215, *q*_110_ = 0.244, *q*_111_ = 0.308, *q*_200_ = 0.345, *q*_210_ = 0.386, *q*_211_ = 0.429, *q*_112_ = 0.454, *q*_220_ = 0.492, *q*_300_ = 0.518, *q*_003_ = 0.577, and *q*_320_ = 0.622 Å^−1^ (Fig. 1a, b). The lattice parameters are *d* = 3.64 nm and *c* = 3.28 nm (Fig. 1c, Supplementary Table 1). This indicates that the inter-dsDNA spacing between parallel dsDNA rods is 3.64 nm. The out-of-plane parameter indicates that the complex has additional order along the dsDNA axis, with a spacing of *c* = 3.28 nm. The melittin monomer (Fig. 1d) has a diameter of ~1.2 nm. The inter-dsDNA spacing of 3.64 nm is larger than that expected from a single melittin molecule at bridging or centered high-symmetry sites (Fig. 1e). To better understand why this occurs, we conducted molecular simulations on the self-assembly of melittin. Since other AMPs have been shown to self-assemble into diverse nanostructures^27^, we hypothesized that melittin can self-assemble into a cationic coiled-coil protofibril in the presence of anionic dsDNA. Starting from the nuclear magnetic resonance monomer structure of melittin (PDB: 2MLT_A), we modeled a 26mer of melittin self-assembled into a protofibril using TINKER and the AMBER force field (Fig. 2a, Supplementary Methods, Supplementary Figures 2, 3, 4). We obtained an optimized monomer structure within the melittin protofibril. The protofibril has a cross-sectional dimension of 2.7 nm × 2.7 nm, with a pitch between monomers of 0.82 nm (~3.28 nm per turn). Melittin packs into a four-fold symmetric protofibril with hydrophobic residues in the interior, and cationic residues exposed to the exterior. We constructed an 8 × 8 square lattice of dsDNA rods with the energy-minimized melittin superhelices (Fig. 2b, c). CRYSOL was used in an iterative process to refine the molecular model of the melittin-dsDNA lattice structure based on the SAXS data. Calculated diffraction peak positions from the energy-minimized structure (Fig. 2b, c) reproduce the scattering peaks seen in the experimental SAXS data (*q*_100_ = 0.168, *q*_001_ = 0.204, *q*_110_ = 0.246, *q*_111_ = 0.302, *q*_200_ = 0.344, *q*_210_ = 0.384, *q*_211_ = 0.431, *q*_112_ = 0.457, *q*_220_ = 0.497, *q*_300_ = 0.525, *q*_003_ = 0.578, and *q*_320_ = 0.621 Å^−1^) (Fig. 2d). Formation of the AMP protofibril makes the large observed inter-dsDNA spacing possible in melittin-dsDNA complexes.

### LL37 organizes dsDNA into a two-dimensional columnar square lattice

**Fig. 3:**
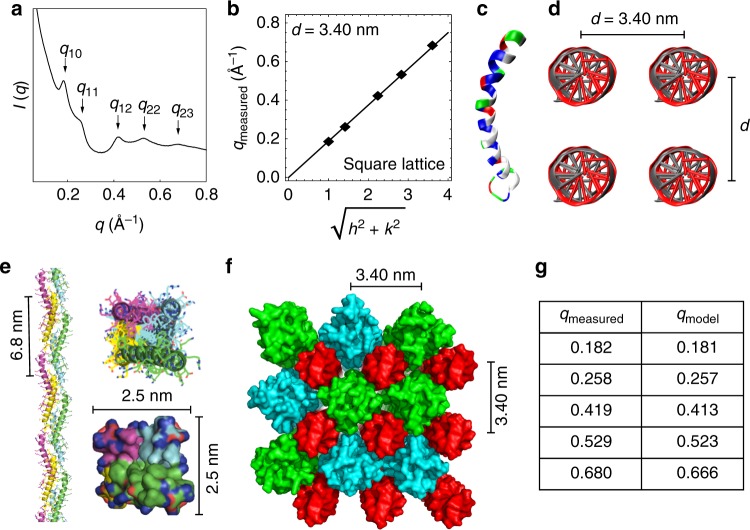
Structure of LL37-double-stranded DNA (dsDNA) complexes from small-angle X-ray scattering (SAXS) and molecular simulations. **a** Representative SAXS data from an isoelectric LL37-dsDNA complex. All observed reflections are labeled with their respective Miller indices (*hk*). **b** Two-dimensional square lattice reflections (*q*_*hk*_) from **a** are fitted to $q_{hk} = \frac{{2\pi }}{d}\sqrt {h^2 + k^2}$, yielding a lattice parameter of *d* = 3.40 nm (*R*^2^ = 0.999). **c** Structure of the LL37 monomer (PDB: 2K6O^59^). Cationic residues are colored in blue and hydrophobic residues are colored in white. **d** Schematic of the LL37-dsDNA square columnar lattice with an inter-dsDNA spacing of *d* = 3.40 nm. The average domain size of the LL37-dsDNA lattice is *L* ~ 16.1 nm. **e** Side and top views of the self-assembled LL37 protofibril structure formed in the presence of dsDNA. The protofibril has rough cross-sectional dimensions of 2.5 nm × 2.5 nm. The helical pitch of the protofibril is 6.8 nm per turn. **f** Top view of a 3 × 3 LL37-dsDNA square lattice from molecular dynamics simulations. Columnar dsDNA is colored red, and the LL37 protofibril is colored green (N to C terminus polarity) and teal (C to N terminus polarity). Random polarity of the LL37 protofibril is assumed. The inter-dsDNA spacing from the molecular model is 3.4 nm, which agrees with the inter-dsDNA spacing predicted from SAXS. **g** There is good agreement between the observed structure factor peaks from SAXS with peaks recovered from theoretical X-ray scattering of the lattice model in **f** from CRYSOL

LL37 is a human ɑ-helical AMP that kills bacteria by forming pores in their cell membranes^31^. However, LL37 is also central to the pathogenesis of autoimmune diseases^13^. Whereas melittin has a proline kink, LL37 has a glycine kink. The isoelectric LL37-dsDNA complex forms a two-dimensional (2D) square columnar lattice with Bragg reflections at *q*_10_ = 0.182, *q*_11_ = 0.258, *q*_12_ = 0.419, *q*_22_ = 0.529, and *q*_23_ = 0.680 Å^−1^, corresponding to a lattice parameter of *d* = 3.40 nm (Fig. 3a, b, Supplementary Table 1). This indicates that the spacing between parallel dsDNA rods is 3.40 nm in this complex. However, the diameter of LL37 is ~1.2 nm (Fig. 3c), which is too small to explain the observed inter-dsDNA spacing from SAXS. X-ray analysis of the LL37-DNA complex in human serum yields a similar result (Supplementary Figure 5). We hypothesize that LL37 assembles into a cationic protofibril that interacts with anionic DNA to form a square columnar lattice (Fig. 3d). We conducted molecular dynamics simulations in a similar manner to the melittin-dsDNA complex to develop a physical model of LL37 self-assembly (Supplementary Methods, Supplementary Figures 2, 3, 4). Starting from the crystal structure of LL37 (PDB: 2K6O), we optimized the structure of an LL37 26mer with multiple rounds of energy minimization and molecular simulation. The final protofibril structure yields four LL37 per helix turn, with a pitch of 6.8 nm and cross section of 2.5 nm × 2.5 nm (Fig. 3e). Similar to melittin, the curved, amphipathic nature of LL37 allows for favorable interactions between the hydrophobic faces of adjacent LL37 in the interior of the protofibril, allowing for outward exposure of the cationic residues on opposite faces. The cationic backbone of the protofibril can then coordinate four dsDNA rods simultaneously, forming a square lattice. Next, we create a full LL37-dsDNA square lattice model by superimposing 3 × 3 dsDNA rods coordinated with multiple LL37 protofibrils (Fig. 3f). We observe that the LL37-dsDNA lattice from simulation has an inter-dsDNA spacing of 3.40 nm, which agrees well with the measured value from SAXS. Using CRYSOL, we observe good correspondence between the major peaks observed in the SAXS diffraction spectrum and the predicted peaks from the atomic structure of simulated lattice (*q*_10_ = 0.181, *q*_11_ = 0.257, *q*_12_ = 0.413, *q*_22_ = 0.523, and *q*_23_ = 0.666 Å^−1^) (Fig. 3g). Note that although a 3D LL37-DNA tetragonal lattice was predicted based on molecular modeling, a cognate 2D square lattice at the same inter-DNA spacings was observed experimentally, likely due to weaker ordering along the protofibril axis compared to the in-plane ordering. Furthermore, we studied effects of various structural parameters on the diffraction pattern. We varied the direction distribution of the AMP superhelices (helical axis C-to-N vs. helical axis N-to-C) and the phase distribution of protofibrils in the lattice (rotation around the superhelical axis). We found that for both the LL37-dsDNA and melittin-dsDNA complexes, these parameters do not strongly impact the observed inter-dsDNA spacing, indicating that these structures are likely at equilibrium.

### Buforin condenses dsDNA into a disordered columnar structure

**Fig. 4:**
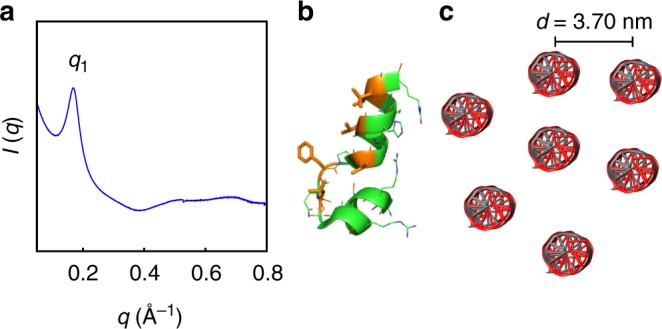
Structure of buforin-double-stranded DNA (dsDNA) complexes. **a** Representative small-angle X-ray scattering (SAXS) data of an isoelectric buforin-dsDNA complex. One main diffraction peak is observed at *q*_1_ = 0.170 Å^−1^ with weak broad features at higher *q*, consistent with a disordered columnar structure with short-ranged order. **b** Structure of the buforin monomer (taken from the H2A histone, PDB: 4KGC^60^). See Supplementary Discussion for details regarding the relationship between buforin and H2A. **c** Illustration of the buforin-dsDNA columnar lattice with average inter-dsDNA spacing of *d* = 3.70 nm

To determine whether the size of the hydrophobic face influences the formation of square columnar DNA lattices with the optimal inter-DNA spacing for TLR9 activation, we chose to study buforin. Compared to melittin and LL37, buforin has similar net charge and charge density, but has a much smaller hydrophobic face (~80°). Unlike LL37 and melittin, buforin is not a full ɑ-helix. The helical portion distal to the proline kink is a random coil. Instead of exhibiting well-defined diffraction peaks that index to a 2D columnar lattice, SAXS data from buforin-dsDNA complexes revealed a single diffraction feature at *q*_1_ = 0.170 Å^−1^ with weaker broad scattering at higher *q*, consistent with a disordered or amorphous structure with an estimated inter-dsDNA spacing of *d* = 3.70 nm (Fig. 4a). This result is interesting since buforin has sufficient charge density to condense DNA. Since buforin has a hydrophobic face that subtends <90° (Fig. 4b), we hypothesized that it cannot stabilize the 360° hydrophobic core in a protofibril with four-fold coordinated helices due to suboptimal interior hydrophobic interactions. Instead, buforin likely forms multimers with various coordination numbers (e.g. 3–5) that give dsDNA columnar lattices with short-ranged order (Fig. 4c).

### AMP-dsDNA complexes amplify inflammation by binding to TLR9

**Fig. 5:**
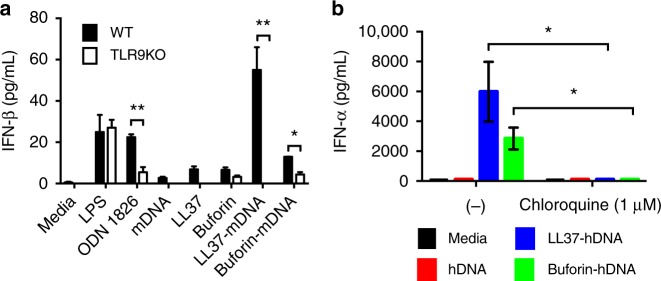
LL37-double-stranded DNA (dsDNA) and buforin-dsDNA complexes induce type 1 interferon (IFN) production in a TLR9-dependent manner in mouse macrophages and human plasmacytoid dendritic cells (pDCs). **a** To measure the ability of AMP-dsDNA complexes to activate TLR9, complexes were incubated with wild-type (WT) and TLR9-knockout (TLR9KO) murine macrophages. IFN-β production was measured and quantified from supernatants using a Bio-ELISA. Mouse genomic dsDNA was used to generate the complexes. Data show TLR9-dependent activation of WT macrophages by LL37-dsDNA and buforin-dsDNA complexes, with minimal to no activation with peptides or dsDNA alone. As a positive control, mouse-specific TLR9 agonist ODN 1826 was used. Both WT and TLR9KO cells responded to the TLR4 agonist lipopolysaccharide (LPS) (*n* = 3, *p < 0.05, **p < 0.01). **b** Similarly, AMP-dsDNA complexes were incubated with human pDCs in the absence or presence of chloroquine (1 µM), and IFN-ɑ production was quantified with enzyme-linked immunosorbent assay (ELISA). Data show that pDCs potently release IFN-ɑ in a TLR9-dependent manner in response to LL37-dsDNA and buforin-dsDNA complexes, and this response is attenuated in the presence of chloroquine, which inhibits endosomal acidification and TLR specific signaling (*n* = 2, *p < 0.05). Error bars denote s.e.m. Group comparisons were calculated using pairwise two-tailed *t*-tests

To investigate the ability of melittin-, LL37-, and buforin-dsDNA complexes to activate TLR9, we incubated pre-mixed AMP-dsDNA complexes with murine wild-type (WT) and TLR9-knockout (TLR9KO) macrophages and measured subsequent IFN-β production (Fig. 5a). In a parallel assay, we also stimulated TLR9-expressing human pDCs in the absence and presence of chloroquine and measured subsequent IFN-ɑ production (Fig. 5b). LL37 and buforin were mixed with mouse genomic dsDNA (mdsDNA) in the macrophage experiments and human genomic dsDNA (hdsDNA) in the pDC experiments. Melittin was unable to induce cytokine production because it was highly cytotoxic to immune cells, consistent with previous observations from the literature^32^ (Supplementary Figure 6). In both assays, we observed significant levels of TLR9-dependent type I IFN production in response to both LL37-dsDNA (*p* < 0.01 macrophage, *p* < 0.05 pDC) and buforin-dsDNA complexes (*p* < 0.05 macrophage, *p* < 0.05 pDC) (Fig. 5a, b). In the murine macrophage system, both complexes produced IFN levels comparable to or greater than stimulation with lipopolysaccharide (LPS; TLR4 agonist) or ODN 1826 (TLR9 agonist). No significant activation was observed by peptides alone or mdsDNA alone. In the human pDC system, both LL37-dsDNA and buforin-dsDNA complexes produced significant TLR9-dependent IFN production compared to controls. The presence of 1 μM chloroquine, an endosomal acidification inhibitor, abrogates all TLR9-dependent signaling. These data confirm that both LL37 and buforin drive immune cell activation in a TLR9-dependent manner. Genomic dsDNA by itself is non-immunogenic, but complexation with AMPs enables breakdown of immune tolerance^10^. The consistency in results between the murine and human systems suggests that this phenomenon is general and reinforces the idea that the crystallinity of these AMP-dsDNA complexes are important determinants governing amplified TLR9 activation. We observed that the LL37-dsDNA complex activates 3–5× more strongly than the buforin-dsDNA complex. The average domain size for the ordered LL37-dsDNA nanocrystalline complex is 16.1 nm, whereas buforin-dsDNA complexes are disordered, with an estimated domain size of 4.3 nm. Note that SAXS measurements are performed on precipitated nanocrystals, and that domain sizes will be limited by the size of the nanocrystalline complexes. These observations on complexes that activate TLR9 are consistent with the conjecture that more crystalline complexes (e.g. well-ordered complexes with defined inter-DNA spacings and a larger number of DNA ligands) lead to greater potential TLR9 activation due to multivalent binding to TLR9.

### Trade-offs in AMP immunomodulatory and membrane activity

The observation that melittin has poor IFN-inducing abilities via TLR9 is an intriguing one, especially since melittin-dsDNA complexes exhibit an optimal inter-dsDNA spacing similar to that of the LL37-dsDNA complex and exhibit good crystallinity (domain size ~ 62.4 nm). Both melittin and LL37 facilitate self-assembly into a protofibril in the presence of dsDNA (Figs. 1–3). However, the hydrophobic face of melittin (~180°) is larger than that of LL37 (~120°); such increased degrees of hydrophobicity in AMPs often correspond to increased nonspecific membrane activity. In the present context, this is not inconsistent with the increased toxicity of melittin to immune cells relative to that of LL37^33^.

**Fig. 6:**
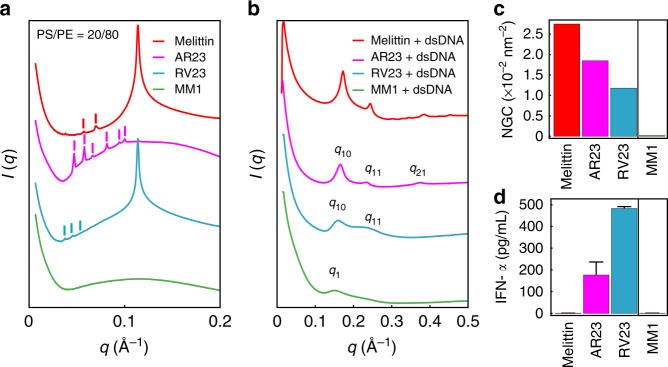
Systematic reduction of the membrane activity of melittin in peptides with conserved double-stranded DNA (dsDNA)-binding activity corresponds with a recovery of TLR9-activating ability. **a** Small-angle X-ray scattering (SAXS) spectra from PS/PE = 20/80 model membranes incubated with melittin, AR23, RV23, and MM1 at *P*/*L* = 1/2. Melittin, AR23, and RV23 induce *Pn3m* cubic phases in model membranes, while MM1 does not induce appreciable changes compared to controls (Supplementary Figure 7). Correlation peaks corresponding to assigned reflections for the *Pn3m* cubic phases are labeled for melittin, AR23, and RV23. **b** SAXS spectra corresponding to the structures of melittin-, AR23-, RV23-, and MM1-DNA complexes. The melittin-DNA SAXS data are reproduced here from Fig. 1 for ease of comparison. Higher-order reflections for the AR23 and RV23 square lattices are labeled. AR23 and RV23 form square columnar lattices with dsDNA (similar to melittin and LL37), while MM1 forms a disordered columnar arrangement. **c** The absolute value of average negative Gaussian curvature (NGC) is calculated from the lattice parameter (*a*) of the cubic phases measured in **a**. Compared to melittin, AR23 and RV23 induce decreasing amounts of NGC in membranes, while MM1 does not induce NGC at all. **d** Measured TLR9-specific interferon (IFN)-ɑ production (pg mL^−1^) from human pDCs induced by AMP-dsDNA complexes. Systematic reduction of NGC-inducing ability in melittin-related peptides AR23 and RV23 (**b**) correlate with a recovery in ability to induce IFN-ɑ from pDCs. MM1 is unable to induce NGC or IFN-ɑ production from pDCs. Error bars denote s.e.m.

To better understand the complex relationship between membrane activity, dsDNA-binding, and TLR9 activation by ɑ-helical AMPs, we selected three melittin-inspired peptides to study, AR23, RV23, and MM1, in addition to melittin itself. AR23 and RV23 are 23-amino acid ɑ-helical structural analogs of melittin, with both containing a proline hinge similar to melittin. Both AR23 and RV23 possess reduced hemolytic and cytotoxic activity compared to melittin^34,35^, and have either reduced charge or reduced overall hydrophobicity, respectively. MM1 is a melittin point mutant with several modified amino acids, including removal of the proline hinge and reduced overall hydrophobicity. Melittin, AR23, RV23, and MM1 were incubated with PS/PE = 20/80 model membranes and the induced negative Gaussian curvature (NGC), the type of curvature topologically required for membrane permeation^36–42^, was measured using SAXS (Fig. 6a, c). In separate experiments, AMP-dsDNA complexes using the peptides were solved using SAXS (Fig. 6b). The TLR9-activating abilities of the AMP-dsDNA complexes were determined via immune stimulation experiments with human pDCs (Fig. 6d).

Melittin, AR23, and RV23 induced cubic phases rich in NGC in model membranes, as evidenced by peaks satisfying the *Pn3m q*-ratios of √2:√3:√4:√6:√8:√9 (Fig. 6a). In contrast, MM1 did not induce cubic phases (membrane only controls are shown in Supplementary Figure 7). The quantity of NGC induced by the peptides in identical membranes is calculated from the lattice parameters (*a*) of the *Pn3m* cubic phases measured, according to the formula ⧼*k*⧽ = 2*πχ*/*A*_0_*a*^2^ (*χ* = −2 and *A*_0_ *=* 1.919 for *Pn3m*). Melittin generated the largest amount of NGC ⧼*k*_melittin_⧽ = −2.75 × 10^−2^ nm^−2^, corresponding to a lattice parameter of *a*_melittin_ = 15.4 nm. In contrast, AR23 and RV23 induced reduced quantitative amounts of NGC from the same membranes, with ⧼*k*_AR23_⧽ = −1.85 × 10^−2^ nm^−2^ and ⧼*k*_RV23_⧽ = −1.18 × 10^−2^ nm^−2^ (Fig. 6c, fits in Supplementary Figure 8). Thus, melittin, AR23, RV23, and MM1 are representative examples of ɑ-helical AMPs that span a range of membrane remodeling ability, with melittin being the strongest and MM1 being the weakest.

To determine whether the differences in charge and hydrophobicity of melittin-related AMPs affected their ability to bind to DNA, we measured the structures of AR23-, RV23-, and MM1-DNA complexes with SAXS and compared them to the structure of the melittin-DNA complex (Fig. 6b, fits in Supplementary Figure 9, Supplementary Table 1) (melittin-DNA SAXS data are reproduced from Fig. 1 for clarity). AR23 and RV23 form square columnar lattices with dsDNA, in a similar manner to melittin and LL37 (Figs. 1–3). AR23-DNA and RV23-DNA complexes exhibit inter-DNA spacings (*d*_AR23_ = 3.78 nm, *d*_RV23_ = 3.87 nm) similar to melittin-DNA (*d*_melittin_ = 3.64 nm) and slightly larger than LL37-DNA (*d*_LL37_ = 3.40 nm) but still in the activating range (Supplementary Table 1). This indicates that despite amino-acid changes leading to reduced membrane activity, AR23 and RV23 retain the ability to organize DNA into a four-fold square lattice. In contrast, MM1-DNA forms a disordered structure with short-ranged order (Fig. 6b). MM1-DNA complexes exhibit more disorder than melittin-, AR23-, RV23-, and LL37-DNA complexes, and have a significantly larger inter-DNA spacing (*d*_MM1_ = 4.19 nm).

Based on these measured inter-DNA spacings, we would predict that AR23-DNA and RV23-DNA complexes possess the ability to induce TLR9-mediated inflammation in pDCs, while MM1 would not. The above complexes were incubated with human pDCs and resulting IFN-ɑ production was measured (Fig. 6d). As expected, AR23- and RV23-DNA complexes both induced greater IFN-ɑ production from pDCs compared to both melittin and MM1-DNA complexes. Interestingly, the increase in TLR9-activating ability in AR23 and RV23 relative to melittin negatively correlates with induced NGC in cell membranes (Fig. 6c, d). For peptides that have conserved DNA-binding and square lattice-forming activity (AR23 and RV23), systematic reduction of NGC-inducing ability corresponds to a recovery in ability to induce IFN-ɑ from pDCs. MM1, however, is not only unable to organize DNA into a square lattice with an inter-DNA spacing in the optimal range but also unable to induce NGC. This is consistent with mutational changes in its sequence that lead to dual abolishment of membrane activity and DNA-binding/TLR9-activating ability. Taken together, these findings suggest that the physiochemical requirements for membrane activity and dsDNA-binding/TLR9 activation of ɑ-helical AMPs are overlapping but distinct. Moreover, trade-offs between these two channels of activity in a given AMP sequence are possible.

## Discussion

One central question is how the existence of these peptide-dsDNA complexes relate to disease states. LL37-DNA complexes are found in vivo in patients with systemic lupus erythematosus^11^. These complexes are formed extracellularly during inflammation and neutrophil cell death (NETosis), and are internalized into phagocytic immune cells, which trigger production of proinflammatory cytokines via TLR9 activation. Furthermore, the presence of LL37-DNA complexes in the serum of lupus patients correlates with increased cytokine production and induce production of autoantibodies to DNA and to AMPs. LL37-mitochondrial DNA complexes were found to be enriched in plasma and atherosclerotic plaques in a mouse model of atherosclerosis; such complexes were also found to induce TLR9-mediated inflammatory responses^43^. Other examples of AMP-DNA complexes relevant to inflammation in autoimmune diseases include IL-26-DNA complexes^44,45^ as well as hBD2-DNA, hBD3-DNA, and lysozyme-DNA complexes^12^. Thus, AMP-DNA complexes are clearly relevant to autoimmune and inflammatory diseases. What is unknown is how complexes between AaHs and dsDNA impact immune outcomes.

AaHs are broadly recognized as membrane-binding peptides. While it has been known that AMPs can be immunomodulatory, it has only been recently discovered that ɑ-helical amphiphilic AMPs form immunologically active complexes with immune ligands^9,28^. In the above experiments, we show that low-symmetry Janus objects such as curved AaHs can self-organize into ordered chaperone protofibrils (cognate to amyloid fibrils^46^) that nucleate the assembly of dsDNA immune ligands into ordered nanocrystalline complexes with well-defined inter-ligand spacings, which in turn amplify cytokine secretion via multivalent binding to TLR9.

In the systems considered here, electrostatic and hydrophobic effects amplify Janus assembly. Clearly, AaHs have too small of a steric diameter to organize the required proinflammatory inter-DNA distances (~3.6 nm) in a given DNA-AaH complex. Aggregation of amphiphiles into spherical micelles is a common mode of assembly, and aggregation of Janus objects with various types of patchiness into complex assemblies has also been well-documented^16,18^. Here the Janus assembly of AaH into four-fold coordinated superhelical protofibrils is driven in part by the creation of an appropriate cationic surface charge density to compensate the anionic charge density of DNA; this effect is pivotal for generating the macromolecular structures optimal for TLR9 activation. In this context, a comparison between melittin and LL37 peptides is telling. Both peptides are amphiphilic and have 35–40% hydrophobic residues (Supplementary Table 2). On the hydrophilic face of the two peptides, the density of the positive-charged residues (lysine or arginine) along the axis are 21 per 3.4 nm (Supplementary Table 3), which matches well to the negative charge density of DNA, 20 per 3.4 nm. This charge compensation allows for maximal entropy gain from counterion release upon binding^47^. Interestingly, melittin has no negatively charged residues while LL37 has ~1.5 e^−^ per 3.4 nm. The negatively charged residues in LL37 contribute to the stabilization of its long helix and will interact with cations (such as Na^+^) in the AMP/DNA lattice. The electrostatic potential surface of melittin and LL37 peptides do not show significant differences as both are dominated by the well-exposed positive residues (Supplementary Figure 10). That the superhelical protofibril architecture can modify its pitch to accommodate AMP subunits with different charge distributions suggests a soft torsional modulus and flexible mechanism of assembly, reminiscent of other forms of electrostatic assembly between macroions^48^. The ability to assemble in this specific manner and the concomitant ability to amplify innate immune responses place constraints on the cationic amino-acid placement, hydrophobic content, and geometric morphology of the constituent AaH. (For example, straight AaHs cannot assemble into protofibrils in this way, so the central glycine in LL37 and proline in melittin are important).

Coming full circle to the canonical function of membrane-active AMPs, it is interesting to compare the structural requirements for membrane permeation and those for immunomodulation via the specific pathway of DNA-binding and TLR9. Our results above suggest that there are trade-offs between the two in a given AMP sequence. Melittin, which is conventionally thought of as a toxin rather than an AMP due to its low selectivity for microbial vs host membranes, forms ordered lattices with DNA, similar to LL37. However, melittin-DNA complexes cannot appreciably induce TLR9 activation despite having a similar inter-DNA spacing to LL37-DNA. By studying melittin mutants and analogs, we find that we can progressively knock in the ability for TLR9 amplification by detuning the melittin sequence away from maximal generation of NGC necessary for membrane permeation. This has implications for the molecular design of multifunctional peptides that are both antimicrobial and immunomodulatory^49,50^.

An understanding of the rules for immunomodulation by peptides can be broadly enabling. Here we show using several prototypical AMPs how AaHs can assemble DNA into an immunoactive nanocrystalline complex that amplifies the TLR9 pathway and illustrate governing rules for this process via AaH sequences capable of different quantitative degrees of immune modulation. From our recent work on TLR3 activation by nanocrystalline AMP-dsRNA complexes^9^, it will not be surprising that more general rules exist.

## Methods

### Preparation of self-assembled peptide-dsDNA complexes

Lyophilized peptides LL37 (LLGDFFRKSKEKIGKEFKRIVQRIKDFLRNLVPRTES), melittin (GIGAVLKVLTTGLPALISWIKRKRQQ), buforin (TRSSRAGLQFPVGRVHRLLRK), MM1 (GIGAVLKALTTGLGALASAIKRKRQQ), AR23 (AIGSILGALAKGLPTLISWIKNR), and RV23 (RIGVLLARLPKLFSLFKLMGKKV) were purchased from Anaspec or Lifetein (≥95% purity by high-performance liquid chromatography (HPLC)) and dissolved in nuclease-free water (Ambion) to 10 mg mL^−1^. For SAXS experiments, monodispersed Lambda dsDNA (New England BioLabs) or *Escherichia coli* genomic dsDNA (Affymetrix) was precipitated and resuspended in 100 mM NaCl + 10 mM HEPES (pH 7.4) to 5 mg mL^−1^. For cell experiments, endotoxin-free human (hdsDNA) and mouse (mdsDNA) genomic self-dsDNA were purchased (Biochain) and used without further purification. Peptide-dsDNA complexes were formed by incubating the peptide with dsDNA (1–5 mg mL^−1^ for SAXS experiments and 10–20 μg mL^−1^ for cell experiments) at specific peptide-to-dsDNA charge ratios (P/dsDNA = 1:4, 1:2, 1:1, 2:1, 4:1). For a particular peptide, the same charge ratios were used in both SAXS and cell experiments. For some experiments, complexes were mixed in the presence of human serum (Sigma-Aldrich, H4522).

### SAXS experiments with peptide-dsDNA complexes

We measured the structural phase diagram of the structures of peptide-dsDNA complexes by incubating peptides (10 mg mL^−1^) with dsDNA (5 mg mL^−1^) at specific charge ratios in microcentrifuge tubes. After thorough mixing and centrifugation, precipitated complexes are hermetically sealed in 1.5 mm quartz capillaries (Hilgenberg GmbH, Mark-tubes). SAXS experiments were performed at Stanford Synchrotron Radiation Lightsource (SSRL, Beamline 4-2) using monochromatic X-rays with an energy of 9 keV. A Rayonix-MX225-HE detector (pixel size 73.2 μm) was used to measure the scattered radiation. Independent identical samples were prepared and measured over multiple time points to ensure consistency. 2D powder diffraction patterns were integrated using the Nika 1.74^51^ package for Igor Pro 6.37 and FIT2D^52^. SAXS data were analyzed by plotting integrated scattering intensity against the momentum transfer *q* using Mathematica. Peak positions were measured by fitting diffraction peaks to a Lorentzian. Structures of complexes were solved by calculating ratios between the *q*-positions of all measured peaks and comparing them with the permitted reflections for specific structures. The lattice parameter(s) of each phase were calculated by linear regression through points corresponding to measured and theoretical peaks. The lattice parameter *d* indicates the inter-dsDNA spacing between dsDNA columns. For a square columnar lattice, $q_{hk} = \frac{{2\pi }}{d}\sqrt {h^2 + k^2}$. For a tetragonal columnar lattice, $q_{hkl} = 2\pi \sqrt {\frac{{h^2 + k^2}}{{d^2}} + \frac{{l^2}}{{c^2}}}$, where *c* is the out-of-plane lattice parameter along the dsDNA axis. For a columnar lattice with short-ranged order, the inter-dsDNA spacing *a* is estimated from the first peak position by the formula *d* *=* 2*π*/*q*_1_.

### Domain size calculations

To calculate the domain size of the nanocrystalline self-assembled polycation-dsDNA complex, we fitted the first Bragg peak of each SAXS spectrum. From this, we obtained the peak width, the average domain size (*L*), and the number of repeat units (*m*) in each complex. To obtain the domain size, we approximated the structure factor peaks as squared-Lorentzians. The form of the equation used for the fit was

$$S\left( q \right) = \frac{{h^3}}{{4\pi \left( {\left| {q - q_1} \right|^2 + \left( {\frac{h}{2}} \right)^2} \right)^2}}$$

where *q*_1_ is the location of the first peak and *h* is the peak width^28^. The experimental SAXS data were background-subtracted, and the first peak for each complex was fitted using nonlinear least-squares regression in Mathematica. The extracted value for peak width *h* can be related to the average linear domain size *L* using Warren’s approximation^53^. The domain size is related to *h* via the equation^54^

$$L = \frac{{\left( {8\pi } \right)^{\frac{1}{2}}}}{{\frac{h}{2}}}$$

We assume that a typical bundle has an area proportional to *L*^2^. To calculate the number of repeat units in each complex *m*, we estimated this by dividing the linear domain size *L* by the measured inter-dsDNA spacings *d* (*m* = *L*/*d*).

Diffraction from disordered anisotropic systems can in principle be complex. We consider disordered bundles as follows. The occurrence of a single broad diffraction feature in *S*(*q*) suggests a disordered system with short-ranged exponential decay of positional correlations. Here *S*(*q*) has a Lorentzian form. After powder averaging over all solid angles, we approximate the domain size as:

$$L = \frac{{2^{\frac{1}{2}}}}{{\frac{h}{2}}}$$

### Macrophage stimulation experiments

To validate the ability of peptide-dsDNA complexes to activate TLR9, we stimulated WT and TLR9KO BL6 mouse macrophages in vitro and measured subsequent IFN-β production using a Luciferase Bio-ELISA. Peptide-dsDNA complexes were generated as described above, using mouse genomic self-dsDNA. Complexes were incubated at room temperature prior to stimulation. Complexes were generated by mixing 2 μg of mdsDNA (BioChain) with 10 μg of peptides (e.g. LL37 or buforin) in 40 μL of nuclease-free water (Ambion) and then diluted into 200 μL of media for cell stimulation (final concentrations: 10 μg mL^−1^ mdsDNA and 50 μg mL^−1^ peptide).

Positive controls for IFN production include a TLR9-positive control (ODN 1826, Invivogen, 10 μg mL^−1^) and a TLR4-positive control (LPS, Sigma-Aldrich, 200 pg mL^−1^). Negative controls included the peptides alone and genomic dsDNA alone at the same concentrations as used for the immune complex stimulations (Fig. 5b).

WT and TLR9KO macrophages were grown to confluency in RPMI + bovine growth serum (BGS) + penicillin/streptomycin (Pen/Strep) + l-glutamine (Gibco), washed, and seeded into 96-well microplates with a final concentration of 10^5^ cells per well. Complexes were added to WT and TLR9KO cells, diluted to a final volume of 150 μL, and incubated at 37 °C for 10 h. Supernatants were removed and frozen at −80 °C overnight. All stimulations were done in triplicate.

### IFN-β bioassay

IFN-β bioassay was carried out similarly to prior work^55^. Supernatants were assayed for type I IFN production (IFN-β) using ISRE-L929 IFN reporter cells (Bio-ELISA system with IFN-β receptor coupled to a luciferase promoter). ISRE-L929 cells were grown to confluency in RPMI + BGS + Pen/Strep + l-glutamine and plated to a final concentration of 10^5^ cells per well in a 96-well microplate. Fifty microliters of supernatant was incubated with ISRE-L929 cells for 7 h at 37 °C. An eight-point standard curve with twofold serial dilutions of known IFN-β concentrations (R&D Systems) was incubated with ISRE-L929 cells at the same time. ISRE-L929 cells are lysed overnight at −80 °C in Passive Lysis Buffer (Promega). Luciferase production in ISRE-L929 is quantified using a Luciferase Assay System (Promega) with a luminometer plate reader (Biotek). Luciferase production was converted to IFN-β production using the calibrated IFN-β standard curve. Peptide-dsDNA complexes that induce a TLR9-specific response produce a significantly larger amount of IFN-β from WT stimulation compared to TLR9KO stimulation (unpaired Student’s *t*-test).

### Lactate dehydrogenase cell death assay

Cytotoxicity of peptides against WT and TLR9KO bone-marrow-derived macrophages were measured using a lactate dehydrogenase (LDH) release assay kit (Promega). Macrophages were grown to confluency in RPMI 1640 + BGS + Pen/Strep + l-glutamine (Gibco), washed, and seeded into 96-well microplates with a final concentration of 10^5^ cells per well. Cells with stimulated by peptide-DNA complexes for 10 h, and the supernatant was removed for LDH quantification. Fifty microliters of supernatant was placed into a fresh 96-well clear-bottom plate, 50 μL of LDH substrate mix was added, and the plate was incubated at room temperature for 2 min under foil. Fifty microliters of stop solution was added, and the plate was centrifuged briefly to remove air bubbles. The plate absorbance was read at 490 nm and LDH release was quantified relative to a maximum lysis control.

### pDC stimulation experiments

Blood buffy coats of healthy donors were obtained from the Interregionale Blutspende SRK, Bern, Switzerland. Peripheral blood mononuclear cells were obtained by Ficoll centrifugation of blood buffy coats. pDCs were then isolated by magnetic separation using the Diamond pDC Isolation kit II (Miltenyi). Purified pDCs were cultured in 96-well round-bottom plates at 5 × 10^4^ per well in 200 μL RPMI 1640 (GIBCO) supplemented with 10% fetal calf serum (Atlanta Biologicals) and Pen/Strep in the presence of LL37-hdsDNA or buforin-hdsDNA complexes. LL37-hdsDNA and buforin-hdsDNA complexes were generated by mixing 2 μg of hdsDNA (BioChain) with 10 μg of LL37 or buforin in 40 μL of nuclease-free water (Ambion) and then diluted into 200 μL of complete medium for cell stimulation (final concentrations: 10 μg mL^−1^ hdsDNA and 50 μg mL^−1^ LL37 or buforin). In some instances, pDCs were pre-incubated with 1 μM chloroquine diphosphate (Sigma) for 15 min before stimulation. The different supernatants were collected after overnight culture and the levels of type I IFN production were measured by enzyme-linked immunosorbent assay (ELISA; human IFN-α pan, Mabtech).

### SAXS experiments with model membranes

Peptides were purchased (Anaspec, Lifetein) and synthesized in high purity ( >95% HPLC) using solid-phase synthesis. Liposomes were prepared as previously described elsewhere^56^. Lyophilized DOPS (1,2-dioleoyl-sn-glycero-3-phospho-l-serine (sodium salt)) and DOPE (1,2-dioleoyl-sn-glycero-3-phosphoethanolamine) from Avanti Polar Lipids were used. Stock solutions were dissolved in chloroform at 20 mg mL^−1^. Mixtures of these lipids were used as a first-order model for bacterial membranes. DOPS and DOPE were mixed at a 1:4 molar ratio (DOPS/DOPE = 20/80). Chloroform was evaporated under N_2_, and the lipids were further dried overnight under vacuum. Dried lipids were resuspended in 100 mM NaCl + 10 mM HEPES at pH 7.4 to a final concentration of 20 mg mL^−1^. Aqueous lipid solutions were incubated overnight at 37 °C. Liposomes were prepared by sonication of resuspended lipids until clear. Monodispersity in size was obtained via extrusion through a 0.2 μm filter.

Before use, peptides are solubilized in nuclease-free pure water. Liposomes (DOPS/DOPE = 20/80) and peptides were mixed at a peptide-to-lipid charge ratio (*P*/*L*) of 1/2 and equilibrated at room temperature overnight. Precipitated peptide-lipid complexes were loaded into 1.5 mm glass quartz capillaries (Mark-tubes, Hilgenberg GmbH) and hermetically sealed. SAXS experiments were conducted at the Stanford Synchrotron Radiation Laboratory (BL 4–2) with monochromatic X-rays of energy 9 keV. Scattered radiation was collected using a Rayonix-MX225-HE detector (pixel size 73.2 μm). The 2D diffraction patterns were integrated using the Nika 1.74^51^ package for Igor Pro 6.37 (Wavemetrics) and FIT2D^52^. For all samples, multiple measurements were taken for consistency. Samples were incubated at 37 °C and centrifuged at 6000 RPM for 20 min before measurement. No changes were observed over multiple experiments and exposures.

SAXS data were analyzed by plotting the integrated scattering intensity *I*(*q*) vs. *q* using Mathematica. To determine the phases present in each sample, the measured peak positions were obtained and their ratios were compared to the permitted reflections for different liquid-crystalline lipid phases (e.g. lamellar, inverse hexagonal, and cubic). Membrane curvature-generating ability of the peptides was determined by calculating the lattice parameter *a* for the measured cubic phases. Cubic phases observed in our experiments belonged to the *Pn3m* space group. Measured *q* positions for the Bragg peak reflections were fitted to the equation *q*_measured_ = 2*π*√(*h*^2^ + *k*^2^ + *l*^*2*^)/*a* where (*hkl*) are the Miller indices. The quantity of induced NGC was calculated as the average NGC per unit cell volume using the equation ⧼*k*⧽ = 2*πχ*/*A*_0_*a*^2^, where *χ* is the Euler characteristic and *A*_0_ is the surface area per cubic unit cell for each phase. Parameter values are *χ* = −2 and *A*_0_ *=* 1.919 for *Pn3m*.

### Statistical analyses

Analysis of all immune stimulation data was done in Mathematica using pairwise two-tailed *t*-tests with a significance level of *α* = 0.05.

### Code availability

Code for molecular modeling will be provided upon reasonable request.

## Supplementary information

Supplementary Information

## Data Availability

Data supporting the findings of this study are available within the Article and its Supplementary Information files and from the corresponding authors upon reasonable request.
