# Supplementary material for: Helical antimicrobial peptides assemble into protofibril scaffolds that present ordered dsDNA to TLR9
Source: Nat Commun. 2019 Mar 4;10:1012. doi: 10.1038/s41467-019-08868-w (PMC6399285; doi:10.1038/s41467-019-08868-w)
Supplement: Supplementary file 1 — Supplementary Information [file 41467_2019_8868_MOESM1_ESM.pdf]

Helical antimicrobial peptides assemble into protofibril scaffolds that present ordered dsDNA to TLR9

Lee et al.

Supplementary Methods

Molecular Modeling and Diffraction Simulations

LL37 and melittin superhelical protofibril modeling

Three parameters were needed to build the superhelical protofibril structure from the monomer: the helical axis, the rotation angle α, and the pitch *c* (Supplementary Figure 2). To obtain a square or near square column, which give a diffraction extinction same as experiment, the rotation angles of both melittin and LL37 were fixed at 90°.

From the diffraction pattern analysis, the periodic length in axis direction of melittin superhelix was 3.28 Å, which was 4 times of the pitch value. *c*(Melittin) =0.82 nm. The following equation was used to estimate the pitch of the LL37 superhelix,

$$c(\mathrm{LL37}) = c(\mathrm{Melittin}) \times \frac{w(\mathrm{LL37})}{w(\mathrm{Melittin})} \times \left[\frac{d(\mathrm{Melittin})}{d(\mathrm{LL37})}\right]^2$$

where, *c* is the pitch, *w* is the molecular weight of monomer, and *d* is the side length of the square cross-section. In this equation, We assume that density of the LL37 lattice and the melittin superhelix are the same. Per helical turn, the volume of the square column is $4 \times d^2 \times c$ and the weight of the column is $4 \times w$. Thus, the density was $w/_{d^2 c}$. If assign *w*(Melittin) = 2.85 kDa, *w*(LL37) = 5.31 kDa, then, *c*(LL37) =1.7 nm.

The helical axis was determined based the amphipathic properties of the AMP monomers. In every 4 residues (about 1 helical turn), there are one or two hydrophobic residues (VILFW) and one or two charged residues (DEKR). PDB structures 2MLT_A [1] and 2K6O [2] were used as the building blocks of the melittin and LL37 superhelices, respectively. Since they both have a hydrophobic and hydrophilic face, "hydrophobic" and "hydrophilic" lines were first determined (Supplementary Figure 3). The "hydrophobic" line denotes the minimum distance to all the hydrophobic side chains, and the "hydrophilic" line denotes the minimum distance to all the charged-residue side chains. Furthermore, these two lines must be parallel. The initial axis of the superhelix was placed in the plane of the two lines and parallel to them, and the distance to the "hydrophilic" line was observed to be 1 nm.

26mer superhelix models were built for both melittin or LL37. Left handed superhelix models exhibited a greater percentage of buried hydrophobic residues than that of the right-handed models. Thus, we hypothesized that the predominant species was the left-handed superhelix. After the initial superhelix structure was constructed, we optimized the structure by several iterations of structure relaxation and helical symmetry reconstruction (Supplementary Figure 4). Energy minimization was first used for structure relaxation. TINKER MINIMIZE program[3] and the AMBER-03[4] force field with GBSA implicit solvent were used for superhelix energy minimization with a threshold gradient 0.5 kcal mol^{-1} $Å^{-1}$. The helical symmetry was reconstructed by averaging the structure of each of the middle 18 monomers, and then repeating the energy minimization

again. This iterative process continued until the root mean square deviation (RMSD) of the superhelix model became smaller than 0.5 Å. Then, a 400 ps explicit-solvent NVT molecular dynamics simulation was carried out for further structure relaxation using the AMBER and AMBER-03 force fields. About 1.0 mol/L NaCl was added after charge neutralization. The system was gradually heated up from 50°K to 300°K at 50°K intervals. After the MD simulation, the helical symmetry was reconstructed. These steps were repeated iteratively until the RMSD thresholds were met. After 4 total MD-reconstruction iteration cycles, the final superhelix column (26mer) structure was obtained. This final model was used for the lattice construction.

AMP-DNA lattice model and the diffraction simulation using CRYSOL

The melittin-DNA and LL37-DNA complexes are square columnar lattices (melittin is tetragonal with an implicit square columnar lattice). The nearest column-column distance is the periodic length ($a = b$). Lattice models were built for melittin (Figure 2) and LL37 (Figure 3).

A GC repeat sequence was used for modeling the B-DNA double helix. The helix has about 10.5 base pairs per turn and the pitch was 34.5 Å. An 8×8 DNA square lattice model was built with a 3.64 (for melittin system) or 3.40 nm (for LL37 system) spacing.

Two kinds of DNA lattice defects were simulated. First, the rotation angles of the DNA in lattice around its axis were randomized. Second, the DNA molecules were not placed at the exact lattice positions, but rather with a random deviation (the maximum deviation was 2 or 4 Å). 100 total lattice models with different random values were built for each AMP-DNA system.

Four kinds of defects for AMP superhelix lattice were simulated. First, the superhelix column is directional. In the lattice, the two column directions (N terminal up or C terminal up) were randomly distributed. Second, the column was rotated around its axis 0-±20° and its diagonal was placed offset to the lattice vector. Third, the columns were randomly deviated (0-2 Å) from the lattice position on the square plane. Fourth, on the axis direction, the lattice is not perfectly ordered. We modeled this by inducing a 0-±c/4 deviation in the center of melittin superhelix columns, and a 0-±c/2 deviation in the center of the LL37 superhelix columns. 100 lattice models with different random values were built for each system.

CRYSOL was used to evaluate the solution scattering from the modeled lattice structure. This program uses multipole expansion of the scattering amplitudes to calculate the spherically averaged scattering pattern and takes into account the hydration shell. The Maximum scattering vector was set to 1.0 $Å^{-1}$ and the number of points in the theoretical curve was set to 1001. Other parameters for CRYSOL were set to default values. The average diffraction curve of the 100 models was used to simulate the lattice diffraction. The parameters of CRYSOL running for DNA lattice were also used for the diffraction calculation of AMP superhelix lattice. The diffraction simulation results of the AMP superhelix lattice (the average of 100 models).

The superhelix lattice model and the DNA lattice model were combined to generate the AMP superhelix/DNA complex lattice model. Comparison of diffraction peak positions between SAXS data and the molecular model is shown in Figure 2 and 3, which shows good agreement.

Supplementary Tables:

Supplementary Table 1: **Structural Parameters of AMP-dsDNA Complexes**. We report crystallinity parameters of the structurally-characterized AMP-dsDNA complexes, including the first peak position *q*, the inter-DNA spacing ($d = 2\pi/q$), the domain size *L*, and the crystalline phase of the structures.

Peptide + DNA	First Peak Position *q* (nm^{-1})	Inter-DNA Spacing *d* (nm)	Peak Widths *h* (nm^{-1})	Domain Size *L* (nm)	Phase
Melittin	1.73	3.64	0.16	62.4	Tetragonal
LL37	1.82	3.40	0.62	16.1	Square
Buforin	1.70	3.70	0.66	4.3	Disordered
AR23	1.65	3.78	0.28	36.2	Square
RV23	1.60	3.87	0.50	20.1	Square
MM1	1.50	4.19	0.89	3.2	Disordered

Supplementary Table 2. **The number and percentage of hydrophobic residues in melittin and LL37 peptides.**

	melittin	LL37
# of hydrophobic residues (ILE, LEU, VAL, PHE, TRP)	10	13
Percentage of hydrophobic residues	10/26=38.5%	13/37=35.1%

Supplementary Table 3. **Charged residues in melittin and LL37 superhelices.**

	melittin	LL37	DNA
# of positive charged residues (ARG or LYS) in peptide	5	11	
# of negative charged residues (ASP or GLU) in peptide	0	5	
Pitch of the superhelix (nm)	3.28/4=0.82	6.8/4=1.7	
Density of positive charges of the superhelix (nm^{-1})	5/0.82=6.10, or **21 per 3.4 nm**	11/1.7 =6.47, or **22 per 3.4 nm**	0
Density of negative charges of the superhelix (nm^{-1})	0	-5/1.7=-2.94	**-20 per 3.4 nm**

Supplementary Figures:

Supplementary Figure 1: **SAXS spectra of DNA Control**. No sharp diffraction features are observed, consistent with typical behavior of uncondensed DNA.

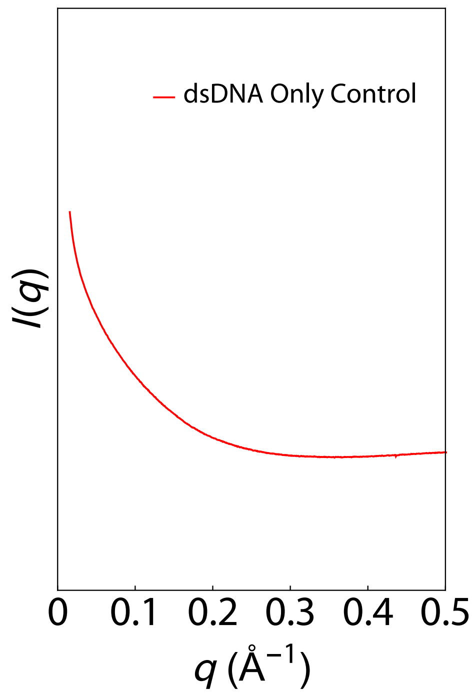

Supplementary Figure 2. **Parameters for building AMP superhelix structure from monomer**. The center-to-center inter-monomer spacing *c* and angle between adjacent monomers α are shown.

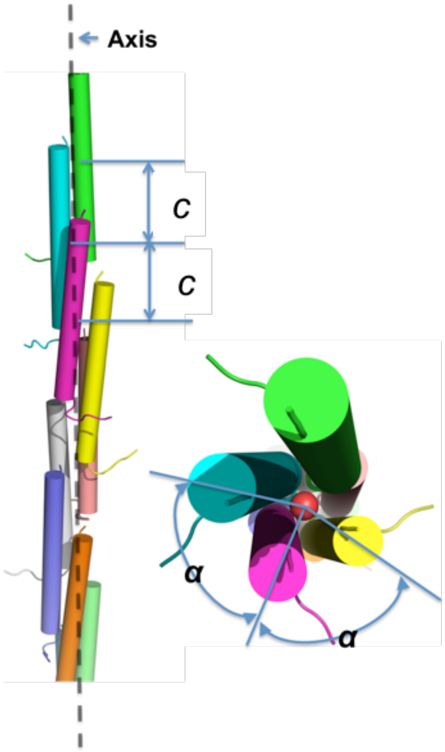

Supplementary Figure 3. **Methods for helical axis determination**. To build the LL37 superhelix, the hydrophobic and hydrophilic lines and the initial axis was determined from the crystal structure. A similar procedure was carried out for constructing the melittin superhelix.

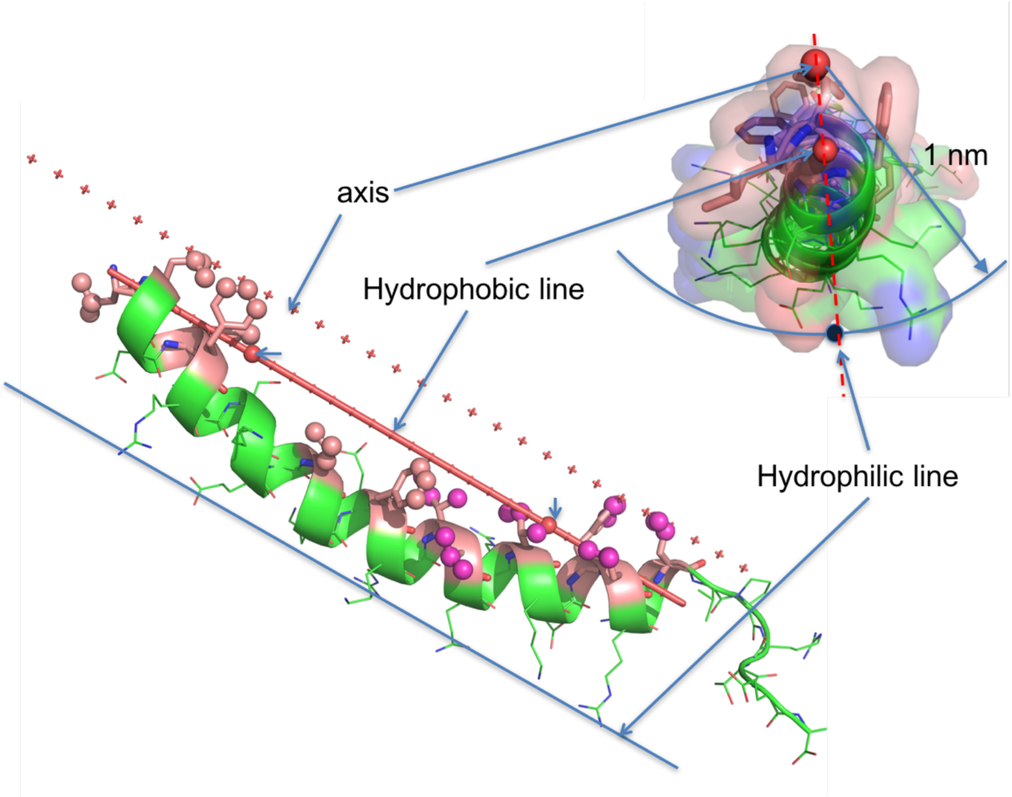

Supplementary Figure 4. **Iterative optimization of the AMP superhelix structure**. To construct the AMP-DNA complexes, multiple rounds of energy minimization and molecular simulation were carried out.

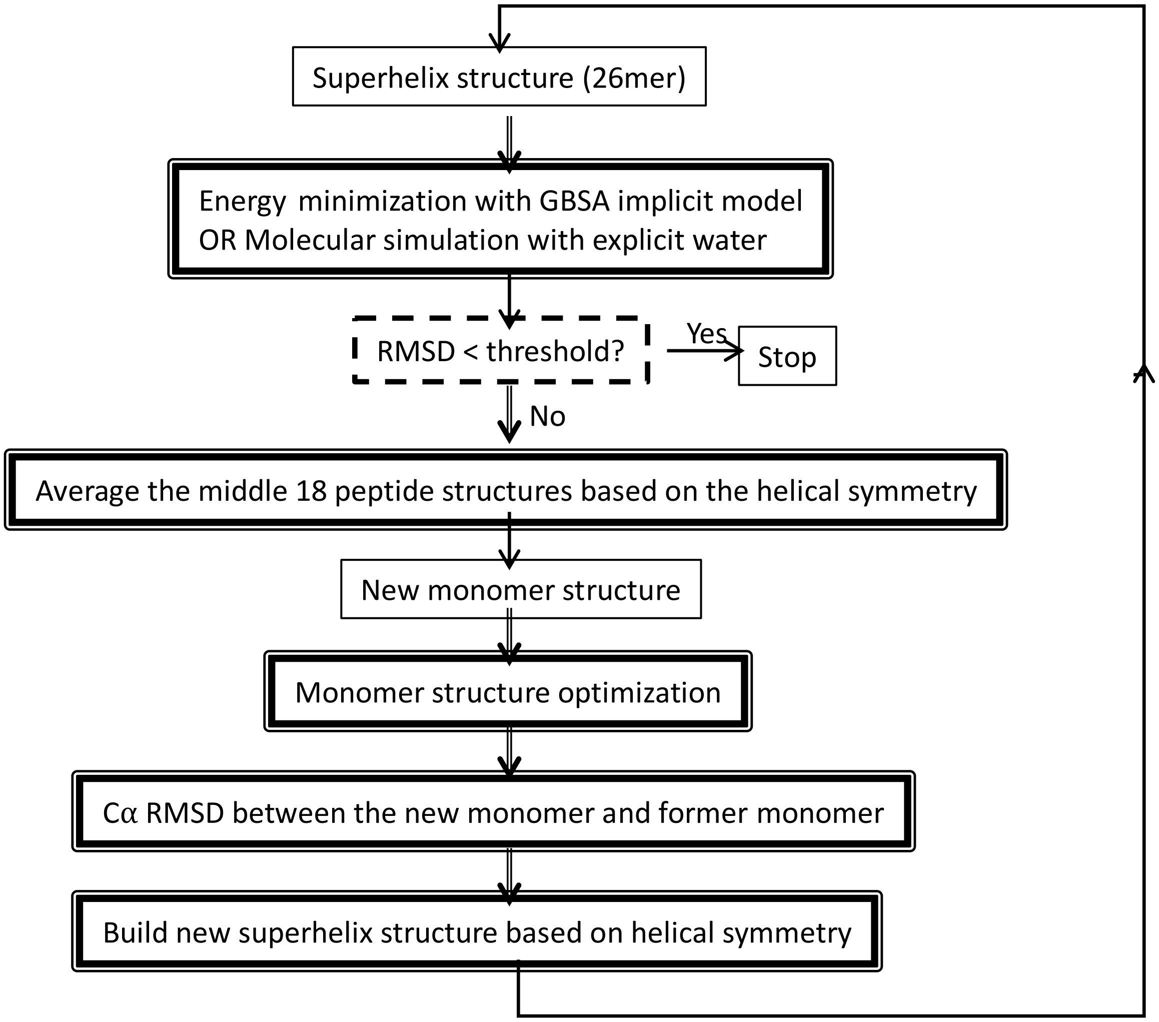

Supplementary Figure 5: **LL37-DNA complexes in human serum**. (A) The LL37-DNA complex in human serum forms a square lattice structure with (B) an inter-DNA spacing of *d* = 3.49 nm, similar to the structure observed for the LL37-DNA complex in physiological salt and buffer and in agreement with computational modeling (Figure 3).

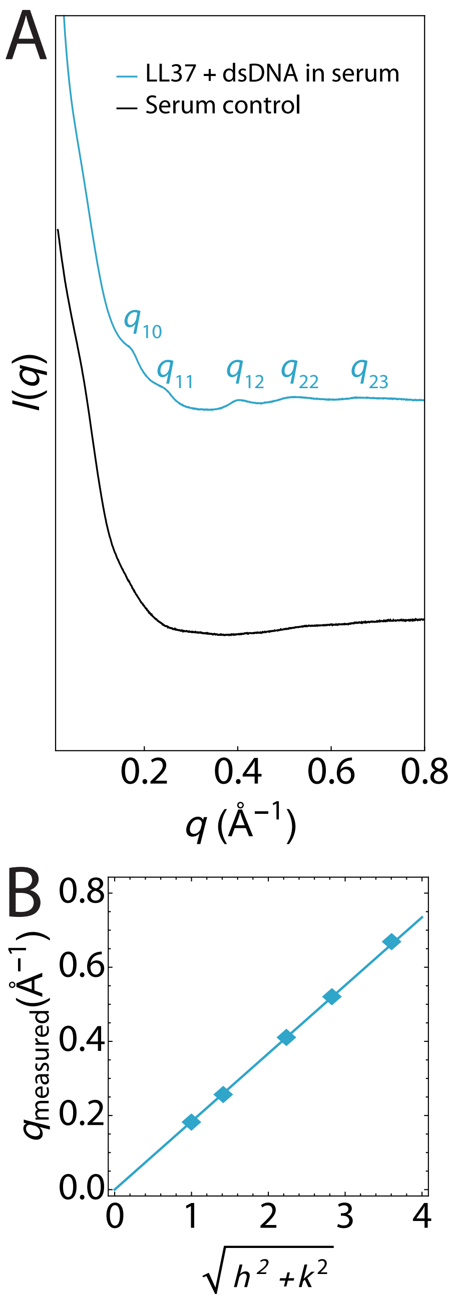

Supplementary Figure 6: **Immune cell cytotoxicity data**. Macrophage cell death induced by the peptide-DNA complexes were quantified using an LDH release assay (Promega) with both the WT and TLR9KO cells. Relative to a maximum lysis control, melittin-DNA complexes killed cells at a much higher rate than the LL37- or buforin-DNA complexes ($p < 0.05$ for both WT and TLR9KO). Error bars denote s.e.m ($n = 3$). Group comparisons were calculated using pairwise two-tailed t-tests.

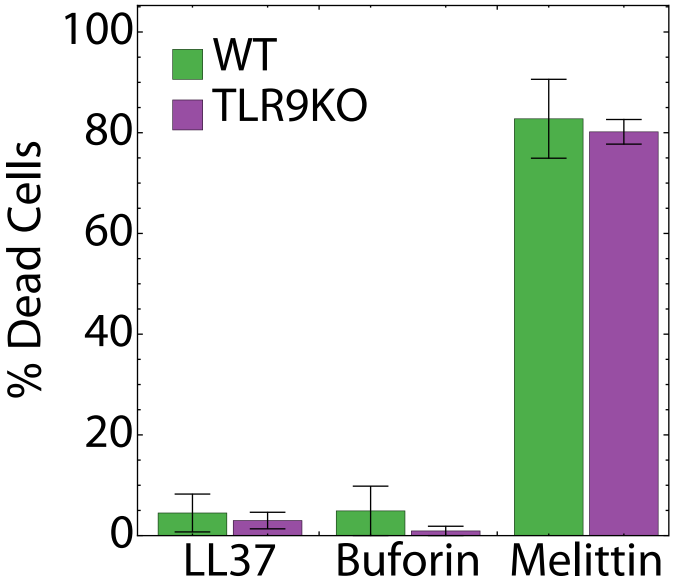

Supplementary Figure 7: **SAXS spectra of SUV Control**. A broad scattering form factor is observed for the control PS/PE = 20/80 SUVs used to probe AMP-membrane interactions.

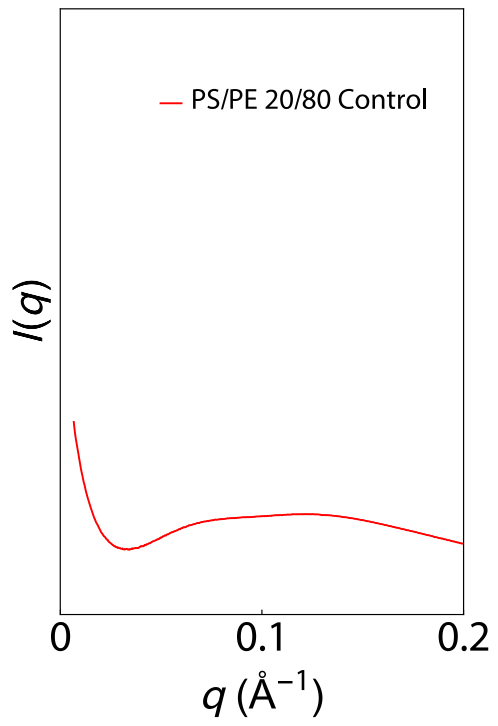

Supplementary Figure 8: **SAXS Lattice Parameter Fits for Membrane Activity of Melittin-related Peptides**. Pn3m cubic phases are induced by melittin (red), AR23 (magenta), and RV23 (teal) peptides in PS/PE = 20/80 membranes. Observed reflections are labeled with their Millier indices (hkl). Lattice parameters *a* are extracted from linear fits, and induced NGC is calculated via the formula $\langle k \rangle = 2\pi\chi/A_0 a^2$, where χ is the Euler characteristic, A_0 is the surface area per cubic unit cell for each phase, and *a* is the lattice parameter. The decreasing slope of the fits from melittin to AR23 corresponds to a decrease in NGC.

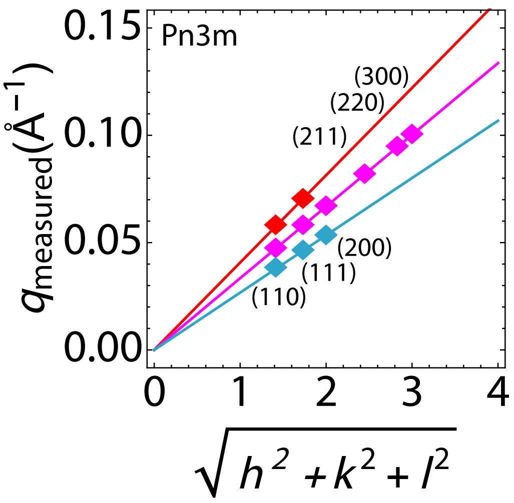

Supplementary Figure 9: **SAXS Lattice Parameter Fits for DNA Complexes with Melittin-related Peptides**. AR23 (magenta) and RV23 (teal) form square columnar complexes with dsDNA. The observed higher order reflections characteristic of the 1:√2:2 ratios are labeled. AR23 and RV23 have lattice parameters of 3.78 nm and 3.87 nm respectively, and the fit demonstrates that they bind DNA similarly.

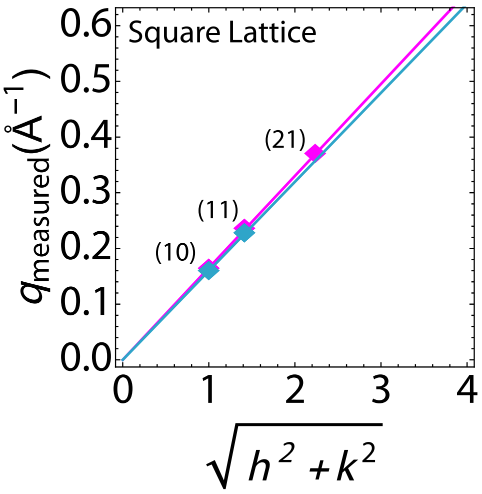

Supplementary Figure 10: **Electrostatic properties of melittin and LL37.** (A) and (C); Charged residues on melittin and LL37. Positively charged residues (lysine and arginine) are colored in blue, and negative residues (glutamic acid and aspartic acid) are colored in red. (B) and (D); Electrostatic potential surface of melittin and LL37 calculated using the MIBPB server [5], which calculates electrostatic potentials with solvation reaction field by solving the Poisson-Boltzmann (PB) equation. The electrostatic surface potential (B or D) is shown in the identical orientation as the cartoon figures (A or C). The surface is colored in red to white, and to blue, indicating the electrostatic potential from -5 to 0, and to +5 kcal/mol/e^-.

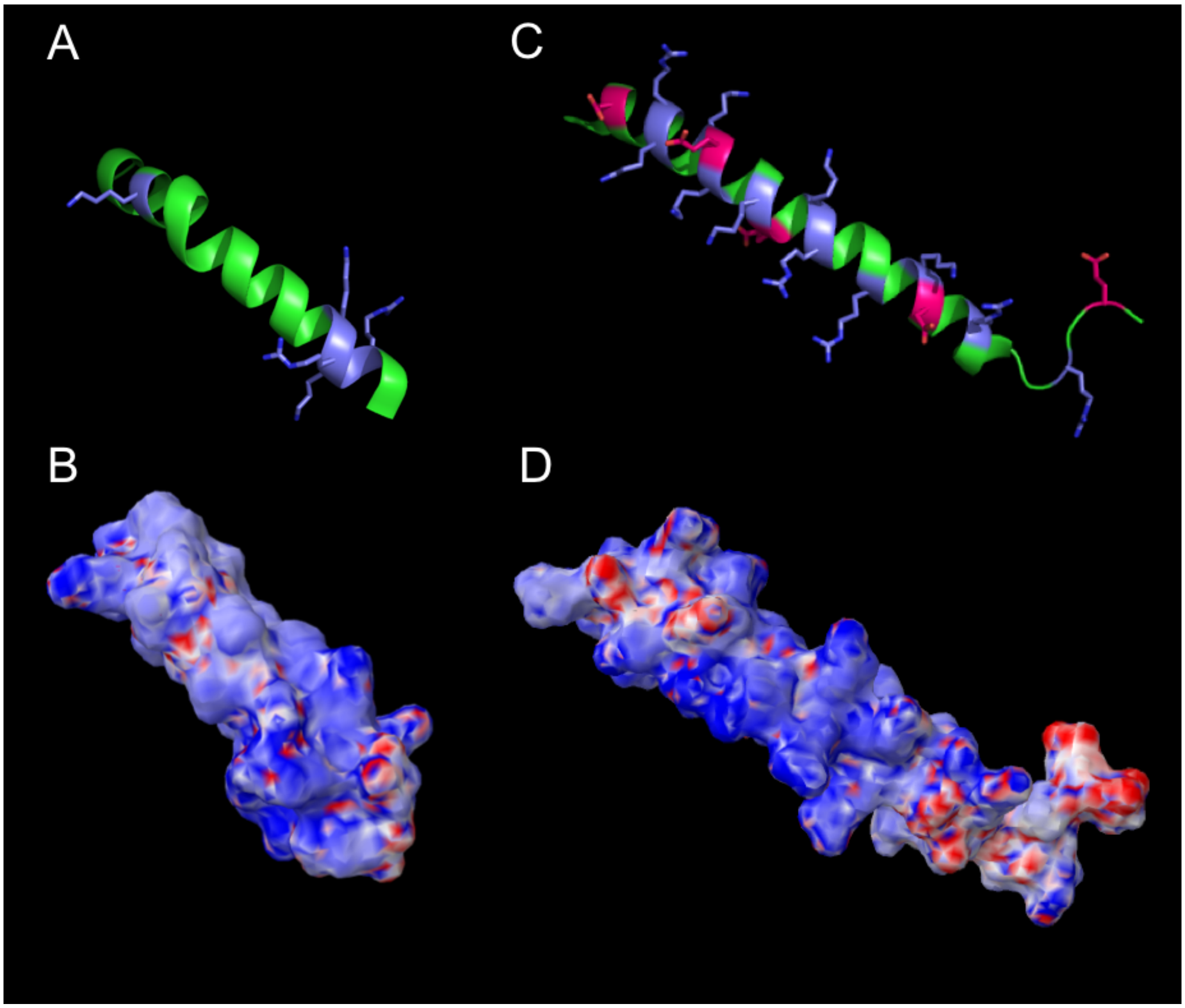

Supplementary Discussion:

The formation of condensed phases of dsDNA are important in biology broad range of biological and biomedical contexts, including the wrapping of dsDNA in chromosomes [6,7] and the delivery of genes into cells [8-10]. Polyelectrolyte chains like dsDNA can collapse into a compact phase from solution as increasing concentrations of polycations are added, driven by the entropy gain of counterion release into bulk solution [11-14]. Condensation of anionic dsDNA by small multivalent cations like hexammine cobalt and spermine and large gene delivery dendrimers have been studied in-depth [15-17], and their structures have been well characterized using X-ray scattering and molecular simulation [18-22]. However, the molecular basis for dsDNA condensation mediated by amphiphilic macroions such as AMPs and other large cationic proteins is complicated by the additional principle of Janus organization.

LL37 (+6 charge at pH 7.4) is a curved α-helix with a glycine at the 14th position [2,23]. This facilitates its antimicrobial properties, allowing it to adsorb onto bacterial membranes and induce pore formation. However, these same properties also allow it to condense dsDNA into nanocrystalline immune complexes. We hypothesized that LL37-like curved α–helical AMPs with sufficient charge density and amphipathicity would be able to condense dsDNA into a lattice with the optimal spacing for TLR9 activation. Typically, curved α–helices are found in soluble peptides and transmembrane helices [24]. The main kink-inducing amino acids are glycine and proline [25] and are typically present toward the center of the α–helix. The presence of proline and glycine can bend helices by 10-20 Å and allow for increased backbone flexibility [26]. We hypothesized that this shape also allows it to self-assemble into a coiled-coiled in the presence of dsDNA. This self-assembly allows for organization of parallel dsDNA rods at a spacing larger than expected for single peptides alone. To look for other candidate peptides that share a similar structural morphology and cationic, amphipathic characteristics, we collated 301 α–helical AMPs from the Antimicrobial Peptide Database [27] (http://aps.unmc.edu/AP/about.php) and analyzed their sequence content. A total of 76/301 AMPs contained a single proline kink, 50 of which were within 5 amino acids of the peptide center. 61/301 AMPs contained a single glycine kink, 41 of which were within 5 amino acids of the peptide center. As a comparison to the glycine-kinked LL37, we selected two well-known α–helical AMPs from this subpopulation with single proline kinks. Melittin (+5 charge at pH 7.4) is 26 amino acids with a proline at the 14th position, and buforin (+6 charge at pH 7.4) is 21 amino acids with a proline at the 11th position. Melittin is a well-studied prototypical α-helical AMP from honey bee venom that also has diverse functions, including cell lysis, antifungal and antibacterial activity [28-30]. Melittin has been reported to bind dsDNA for gene transfer [31], but the structure of the complex has never been studied. Buforin, derived from histone H2A of the Asian toad *Bufo bufo garagrizans*, is structurally homologous to LL37 and melittin with a core α-helical region linked by a proline hinge to an extended helical region with a random coil [32]. Buforin is known to penetrate cell membranes [33] and bind to dsDNA and RNA within bacterial cells, disrupting replication [34].

All three AMPs are highly cationic, with sufficient local surface charge density to bind strongly to DNA. In fact, buforin is a histone fragment and has been shown to bind DNA during antimicrobial function [34], and other AMPs have been shown to bind to DNA [35]. An array of closely spaced cationic charges with a high charge density like those found in these helical AMPs behaves differently from the same number of cationic charges distributed farther apart [36]. For instance, when a linear polyelectrolyte has an inter-charge distance less than the Bjerrum length (<7Å in

water), Manning condensation will induce the formation of a condensed counterion sheath around the polyelectrolyte. Both the cationic AMP and the anionic DNA are expected to have significant association with counterions. As a result, the strongest electrostatic binding occurs when the two binding surfaces have equal and opposite charge density, which will maximize the entropy gain of counterion release into the bulk solution upon binding and charge compensation between the two surfaces [37-39]. This provides a physical basis for AMP-mediated DNA binding and condensation into nanocrystalline structures.

Supplementary References:

1. Terwilliger, T. C. & Eisenberg, D. The structure of melittin. I. Structure determination and partial refinement. *Journal of Biological Chemistry* **257,** 6010–6015 (1982).
2. Wang, G. Structures of human host defense cathelicidin LL-37 and its smallest antimicrobial peptide KR-12 in lipid micelles. *Journal of Biological Chemistry* **283,** 32637–32643 (2008).
3. Ren, P. & Ponder, J. W. Polarizable atomic multipole water model for molecular mechanics simulation. *J. Phys. Chem. B* (2003). doi:10.1021/jp027815
4. Duan, Y. *et al.* A point-charge force field for molecular mechanics simulations of proteins based on condensed-phase quantum mechanical calculations. *J Comput Chem* **24,** 1999–2012 (2003).
5. Chen, D., Chen, Z., Chen, C., Geng, W. & Wei, G.-W. MIBPB: a software package for electrostatic analysis. *J Comput Chem* **32,** 756–770 (2011).
6. Richmond, T. J., Finch, J. T., Rushton, B., Rhodes, D. & Klug, A. Structure of the nucleosome core particle at 7 Å resolution. *Nature* **311,** 532–537 (1984).
7. Schiessel, H. The nucleosome: A transparent, slippery, sticky and yet stable DNA-protein complex. *Eur. Phys. J. E* **19,** 251–262 (2006).
8. Boussif, O. *et al.* A Versatile Vector for Gene and Oligonucleotide Transfer Into Cells in Culture and in-Vivo - Polyethylenimine. *Proc. Natl. Acad. Sci. U. S. A.* **92,** 7297–7301 (1995).
9. Kukowska-Latallo, J. F. *et al.* Efficient transfer of genetic material into mammalian cells using Starburst polyamidoamine dendrimers. *Proc. Natl. Acad. Sci. U. S. A.* **93,** 4897–4902 (1996).
10. Radler, J. O., Koltover, I., Salditt, T. & Safinya, C. R. Structure of DNA-cationic liposome complexes: DNA intercalation in multilamellar membranes in distinct interhelical packing regimes. *Science* **275,** 810–814 (1997).
11. Ray, J. & Manning, G. S. An attractive force between two rodlike polyions mediated by the sharing of condensed counterions. *Langmuir* **10,** 2450–2461 (1994).
12. Schiessel, H. & Pincus, P. Counterion-Condensation-Induced Collapse of Highly Charged Polyelectrolytes. *Macromolecules* **31,** 7953–7959 (1998).
13. Solis, F. J. & la Cruz, de, M. O. Collapse of flexible polyelectrolytes in multivalent salt solutions. *J. Chem. Phys.* **112,** 2030–2035 (2000).
14. Wong, G. C. L. Electrostatics of rigid polyelectrolytes. *Current Opinion in Colloid & Interface Science* **11,** 310–315 (2006).
15. Pollack, L. SAXS studies of ion-nucleic acid interactions. *Annu. Rev. Biophys.* **40,** 225–242 (2011).

16. Allahyarov, E., Gompper, G. & Löwen, H. Attraction between DNA molecules mediated by multivalent ions. *Phys. Rev. E* **69,** 041904–13 (2004).
17. Qiu, X., Andresen, K., Lamb, J. S., Kwok, L. W. & Pollack, L. Abrupt Transition from a Free, Repulsive to a Condensed, Attractive DNA Phase, Induced by Multivalent Polyamine Cations. *Phys. Rev. Lett.* **101,** 228101–4 (2008).
18. Evans, H. M. *et al.* Structural polymorphism of DNA-dendrimer complexes. *Phys. Rev. Lett.* **91,** 075501 (2003).
19. DeRouchey, J., Netz, R. R. & Radler, J. O. Structural investigations of DNA-polycation complexes. *Eur. Phys. J. E* **16,** 17–28 (2005).
20. Ziebarth, J. & Wang, Y. Molecular dynamics simulations of DNA-polycation complex formation. *Biophysj* **97,** 1971–1983 (2009).
21. Prévost, S. *et al.* Colloidal Structure and Stability of DNA/Polycations Polyplexes Investigated by Small Angle Scattering. *Biomacromolecules* **12,** 4272–4282 (2011).
22. Su, C.-J. *et al.* Columnar mesophases of the complexes of DNA with low-generation poly(amido amine) dendrimers. *Biomacromolecules* **10,** 773–783 (2009).
23. Porcelli, F. *et al.* NMR Structure of the Cathelicidin-Derived Human Antimicrobial Peptide LL-37 in Dodecylphosphocholine Micelles. *Biochemistry* **47,** 5565–5572 (2008).
24. Wilman, H. R., Shi, J. & Deane, C. M. Helix kinks are equally prevalent in soluble and membrane proteins. *Proteins: Structure, Function, and Bioinformatics* **82,** 1960–1970 (2014).
25. Deville, J., Rey, J. & Chabbert, M. Comprehensive analysis of the helix-X-helix motif in soluble proteins. *Proteins: Structure, Function, and Bioinformatics* **72,** 115–135 (2008).
26. Jacob, J., Duclohier, H. & Cafiso, D. S. The role of proline and glycine in determining the backbone flexibility of a channel-forming peptide. *Biophysj* **76,** 1367–1376 (1999).
27. Wang, G., Li, X. & Wang, Z. APD3: the antimicrobial peptide database as a tool for research and education. *Nucleic Acids Research* **44,** D1087–93 (2016).
28. Yang, L., Harroun, T. A., Weiss, T. M., Ding, L. & Huang, H. W. Barrel-Stave Model or Toroidal Model? A Case Study on Melittin Pores. *Biophysj* **81,** 1475–1485 (2001).
29. Lee, M.-T., Sun, T.-L., Hung, W.-C. & Huang, H. W. Process of inducing pores in membranes by melittin. *Proc. Natl. Acad. Sci. U. S. A.* **110,** 14243–14248 (2013).
30. Rozengurt, E., Gelehrter, T. D., Legg, A. & Pettican, P. Melittin stimulates Na entry, Na-K pump activity and DNA synthesis in quiescent cultures of mouse cells. *Cell* **23,** 781–788 (1981).
31. Chang-Po Chen, Ji-seon Kim, Erin Steenblock, Dijie Liu, A. & Rice, K. G. Gene Transfer with Poly-Melittin Peptides. *Bioconjug. Chem.* **17,** 1057–1062 (2006).
32. Park, C. B., Yi, K. S., Matsuzaki, K., Kim, M. S. & Kim, S. C. Structure-activity analysis of buforin II, a histone H2A-derived antimicrobial peptide: the proline hinge is responsible for the cell-penetrating ability of buforin II. *Proc. Natl. Acad. Sci. U. S. A.* **97,** 8245–8250 (2000).
33. Kobayashi, S. *et al.* Membrane translocation mechanism of the antimicrobial peptide buforin 2. *Biochemistry* **43,** 15610–15616 (2004).
34. Park, C. B., Kim, H. S. & Kim, S. C. Mechanism of Action of the Antimicrobial Peptide Buforin II: Buforin II Kills Microorganisms by Penetrating the Cell Membrane and Inhibiting Cellular Functions. *Biochem. Biophys. Res. Commun.* **244,** 253–257 (1998).

35. Hsu, C.-H. *et al.* Structural and DNA-binding studies on the bovine antimicrobial peptide, indolicidin: evidence for multiple conformations involved in binding to membranes and DNA. *Nucleic Acids Research* **33,** 4053–4064 (2005).
36. Butler, J. C., Angelini, T. E., Tang, J. X. & Wong, G. C. L. Ion multivalence and like-charge polyelectrolyte attraction. *Phys. Rev. Lett.* **91,** 028301 (2003).
37. Manning, G. S. Limiting Laws and Counterion Condensation in Polyelectrolyte Solutions I. Colligative Properties. *J. Chem. Phys.* **51,** 924–933 (1969).
38. Manning, G. S. Limiting Laws and Counterion Condensation in Polyelectrolyte Solutions II. Self-Diffusion of the Small Ions. *J. Chem. Phys.* **51,** 934–938 (1969).
39. Sanders, L. K. *et al.* Control of electrostatic interactions between F-actin and genetically modified lysozyme in aqueous media. *Proc. Natl. Acad. Sci. U. S. A.* **104,** 15994–15999 (2007).
